# Crystal structure of 1,1′-[selanediyl­bis(4,1-phenyl­ene)]bis­(2-chloro­ethan-1-one)

**DOI:** 10.1107/S2056989015019969

**Published:** 2015-11-14

**Authors:** Hazem Bouraoui, Ali Boudjada, Noudjoud Hamdouni, Youcef Mechehoud, Jean Meinnel

**Affiliations:** a Laboratoire de Cristallographie, Département de Physique, Université Mentouri-Constantine, 25000 Constantine, Algeria; b Laboratoire VAREN, Département de chimie, Faculté des Sciences Exactes, Université Mentouri-Constantine, 25000 Constantine, Algeria; c UMR 6226 CNRS–Université Rennes 1 ‘Sciences Chimiques de Rennes’, Equipe ‘Matière Condensée et Systèmes Electroactifs’, 263 Avenue du Général Leclerc, F-35042 Rennes, France

**Keywords:** crystal structure, organoselenium, selenium, C—H⋯O hydrogen bonds

## Abstract

In the title mol­ecule, C_16_H_12_Cl_2_O_2_Se, the C—Se—C angle is 100.05 (14)°, with the dihedral angle between the planes of the benzene rings being 69.92 (17)°. The average endocyclic angles (Se—C_ar_—C_ar_; ar = aromatic) facing the Se atom are 120.0 (3) and 119.4 (3)°. The Se atom is essentially coplanar with the benzene rings, with Se—C_ar_—C_ar_—C_ar_ torsion angles of −179.2 (3) and −179.7 (3)°. In the crystal, mol­ecules are linked *via* C—H⋯O hydrogen bonds forming chains propagating along the *a*-axis direction. The chains are linked *via* C—H⋯π inter­actions, forming a three-dimensional network.

## Related literature   

For a review of organoselenium chemistry, see: Procter (2001 ▸). For there uses as reagents and inter­mediates in organic synthesis, see: Zade *et al.* (2005 ▸). For their use as inter­mediates in the synthesis of pharmaceuticals, see: Woods *et al.* (1993 ▸), and fine chemicals and polymers, see: Hellberg *et al.* (1997 ▸). For their biological properties, see: Zhu & Jiang (2008 ▸); Anderson *et al.* (1996 ▸); Abdel-Hafez (2008 ▸). For details of how selenium compounds play important roles in protecting the heart, preventing cancer and cardiovascular diseases, see: Yang *et al.* (2005 ▸). For details of how selenium functions as an anti­oxidant in conjunction with vitamin E, see: Ellis *et al.* (1984 ▸). For the synthesis, see: Mechehoud *et al.* (2010 ▸). For related structures, see: Zuo (2013 ▸); Bouraoui *et al.* (2011 ▸).
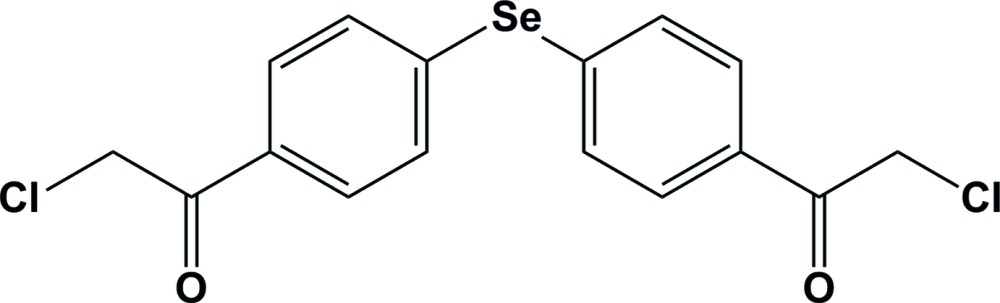


## Experimental   

### Crystal data  

C_16_H_12_Cl_2_O_2_Se
*M* *_r_* = 386.12
Triclinic 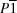

*a* = 7.9664 (7) Å
*b* = 9.2804 (10) Å
*c* = 10.9045 (12) Å
α = 104.612 (9)°
β = 95.886 (8)°
γ = 91.102 (8)°
*V* = 775.14 (14) Å^3^
*Z* = 2
Mo *K*α radiation
μ = 2.77 mm^−1^
*T* = 293 K
0.13 × 0.08 × 0.04 mm

### Data collection  

Oxford Diffraction Xcalibur diffractometer
Absorption correction: multi-scan (*CrysAlis PRO*; Agilent, 2013 ▸) *T*_min_ = 0.519, *T*_max_ = 1.000
8193 measured reflections
4862 independent reflections
2878 reflections with *I* > 2σ(*I*)
*R*_int_ = 0.033

### Refinement  

*R*[*F* ^2^ > 2σ(*F* ^2^)] = 0.056
*wR*(*F* ^2^) = 0.134
*S* = 1.04
4862 reflections
190 parameters
H-atom parameters constrained
Δρ_max_ = 0.59 e Å^−3^
Δρ_min_ = −0.49 e Å^−3^

### 

Data collection: *Xcalibur* (Oxford Diffraction, 2002 ▸); cell refinement: *CrysAlis RED* (Oxford Diffraction, 2002 ▸); data reduction: *CrysAlis RED*; program(s) used to solve structure: *SIR2002* (Burla *et al.*, 2005 ▸); program(s) used to refine structure: *SHELXL2014* (Sheldrick, 2015 ▸); molecular graphics: *PLATON* (Spek, 2009 ▸); software used to prepare material for publication: *SHELXL2014* and *PLATON*.

## Supplementary Material

Crystal structure: contains datablock(s) Global, I. DOI: 10.1107/S2056989015019969/su5221sup1.cif

Structure factors: contains datablock(s) I. DOI: 10.1107/S2056989015019969/su5221Isup2.hkl

Supporting information file. DOI: 10.1107/S2056989015019969/su5221Isup3.cml

**Fig. 1.**
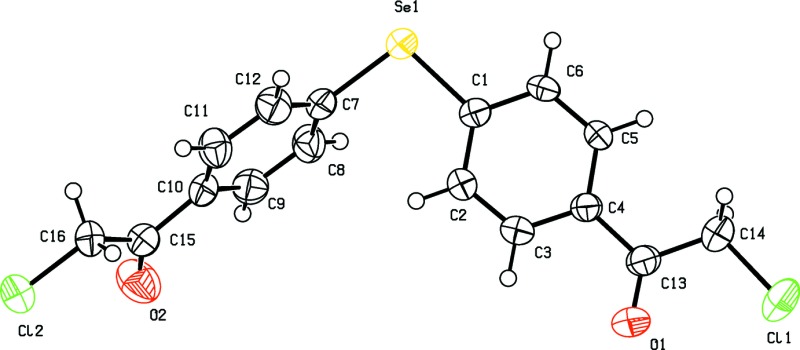
The molecular structure of the title compound, with atom labelling. Displacement ellipsoids are drawn at the 50% probability level.

**Fig. 2.**
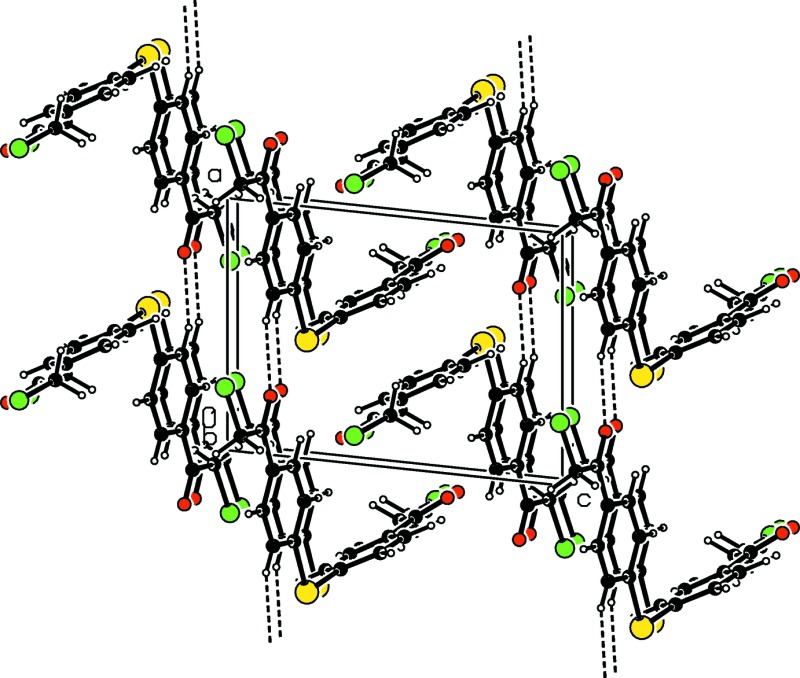
Crystal packing of the title compound, viewed along the b axis. The hydrogen bonds are shown as dashed lines (see Table 1).

. DOI: 10.1107/S2056989015019969/su5221fig1.tif
The mol­ecular structure of the title compound, with atom labelling. Displacement ellipsoids are drawn at the 50% probability level.

b . DOI: 10.1107/S2056989015019969/su5221fig2.tif
Crystal packing of the title compound, viewed along the *b* axis. The hydrogen bonds are shown as dashed lines (see Table 1).

CCDC reference: 1432588

Additional supporting information:  crystallographic information; 3D view; checkCIF report

## Figures and Tables

**Table 1:** Hydrogen-bond geometry (Å, °) *Cg*1 and *Cg*2 are the centroids of benzene rings C1–C6 and C7–C12, respectively.

*D*—H⋯*A*	*D*—H	H⋯*A*	*D*⋯*A*	*D*—H⋯*A*
C6—H61⋯O1^i^	0.93	2.41	3.288 (4)	158
C16—H161⋯*Cg*2^ii^	0.97	2.82	3.611 (4)	140
C16—H162⋯*Cg*1^iii^	0.97	2.92	3.749 (4)	144

Symmetry codes: (i) 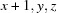; (ii) 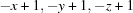; (iii) 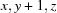.

## supplementary crystallographic information

### S1. Comment

During the last few years, organoselenium chemistry (Procter, 2001) has been the subject of constant scientific inter­est and organoselenium compounds have been used intensively as important reagents and inter­mediates in organic synthesis (Zade *et al.*, 2005). Moreover, organoselenium compounds are of considerable inter­est as anti-cancer (Zhu & Jiang, 2008), anti-oxydant (Anderson *et al.*, 1996), anti-inflammatory, and anti­allergic agents (Abdel-Hafez, 2008) and they are key inter­mediates for the synthesis of pharmaceuticals (Woods *et al.*, 1993), fine chemicals and polymers (Hellberg *et al.*, 1997). Selenium compounds have been found to play important roles in protecting the heart, preventing cancer and cardiovascular diseases (Yang *et al.*, 2005). Because selenium functions as an anti­oxidant it works in conjunction with vitamin E (Ellis *et al.*, 1984).

In the title compound, Fig. 1, the C1—Se1—C7 angle is 100.05 (14) °, similar to the value observed in two very similar compounds, viz. ca 99.47 ° in bis­(4-nitro­phenyl)­selenide (Zuo, 2013), and 99.59 (14)° in bis­(4-acetyl­phenyl) selenide (Bouraoui *et al.*, 2011) where the Se atom lies on a two-fold rotation axis. In the title compound the two benzene rings are inclined to one another by 69.92 (17) °. This is similar to the same angle observed for the 4-nitro­phenyl derivative, ca 63.77 °, but considerabley diffrent to that observed for the acetyl­phenyl derivative, viz. 87.08 (15) °.

In the crystal of the title compound, molecules are linked by C—H···O hydrogen bonds forming chains along the *a* axis direction (Table 1 and Fig. 2). The chains are linked by C—H···π inter­actions (Table 1) forming a three-dimensional structure.

### S2. Synthesis and crystallization

The title compound was prepared according to a method proposed by (Mechehoud *et al.*, 2010). Methyl­ene chloride acyl chloride, ClCH_2_COCl, (36.5 mmol) and anhydrous aluminium chloride 5 g (37.5 mmol, 3 eq) were taken in dry CH_2_Cl_2_ (100 ml). The reaction mixture was cooled to 273-278 K, protected from atmospheric moisture, and stirred continuously for 15 min. A solution of di­phenyl selenide (1) 3 g (1.87 mmol) in CH_2_Cl_2_ was added drop wise over a period of 5 min. The reaction mixture was allowed to reach room temperature gradually and then stirred at this temperature overnight. The solution was then washed with ice water-HCl (80 ml) and extracted with CH_2_Cl_2_. The organic layer was separated and dried (Na_2_SO_4_). Removal of the solvent under reduced pressure afforded the crude product which was recrystallized from ether-petrol to yield 4.2 g of the title compound. Yellow single crystals suitable for X-ray diffraction were obtained by recrystallization from CH_2_Cl_2_.

### S3. Refinement

Crystal data, data collection and structure refinement details are summarized in Table 2. All the H atoms were localized in difference Fourier maps but introduced in calculated positions and treated as riding atoms: C—H = 0.93-0.97 Å with U_iso_(H) = 1.2U_eq_(C).

### Crystal data

**Table:**

C_16_H_12_Cl_2_O_2_Se	*Z* = 2
*M**_r_* = 386.12	*F*(000) = 384
Triclinic, *P*1	*D*_x_ = 1.654 Mg m^−^^3^
Hall symbol: -P 1	Mo *K*α radiation, λ = 0.7107 Å
*a* = 7.9664 (7) Å	Cell parameters from 1842 reflections
*b* = 9.2804 (10) Å	θ = 3.9–27.7°
*c* = 10.9045 (12) Å	µ = 2.77 mm^−^^1^
α = 104.612 (9)°	*T* = 293 K
β = 95.886 (8)°	Needle, colourless
γ = 91.102 (8)°	0.13 × 0.08 × 0.04 mm
*V* = 775.14 (14) Å^3^	

### Data collection

**Table:**

Oxford Diffraction Xcalibur diffractometer	2878 reflections with *I* > 2σ(*I*)
Graphite monochromator	*R*_int_ = 0.033
ω/2θ scans	θ_max_ = 32.0°, θ_min_ = 3.4°
Absorption correction: multi-scan (*CrysAlis PRO*; Agilent, 2013)	*h* = −11→11
*T*_min_ = 0.519, *T*_max_ = 1.000	*k* = −13→13
8193 measured reflections	*l* = −16→11
4862 independent reflections	

### Refinement

**Table:**

Refinement on *F*^2^	Primary atom site location: structure-invariant direct methods
Least-squares matrix: full	Secondary atom site location: difference Fourier map
*R*[*F*^2^ > 2σ(*F*^2^)] = 0.056	Hydrogen site location: inferred from neighbouring sites
*wR*(*F*^2^) = 0.134	H-atom parameters constrained
*S* = 1.04	*w* = 1/[σ^2^(*F*_o_^2^) + (0.047*P*)^2^ + 0.1701*P*] where *P* = (*F*_o_^2^ + 2*F*_c_^2^)/3
4862 reflections	(Δ/σ)_max_ < 0.001
190 parameters	Δρ_max_ = 0.59 e Å^−^^3^
0 restraints	Δρ_min_ = −0.49 e Å^−^^3^

### Special details

**Table:**

Geometry. All e.s.d.'s (except the e.s.d. in the dihedral angle between two l.s. planes) are estimated using the full covariance matrix. The cell e.s.d.'s are taken into account individually in the estimation of e.s.d.'s in distances, angles and torsion angles; correlations between e.s.d.'s in cell parameters are only used when they are defined by crystal symmetry. An approximate (isotropic) treatment of cell e.s.d.'s is used for estimating e.s.d.'s involving l.s. planes.

### Fractional atomic coordinates and isotropic or equivalent isotropic displacement parameters (Å^2^)

**Table:**

	*x*	*y*	*z*	*U*_iso_*/*U*_eq_	
Se1	0.53262 (4)	0.10191 (4)	0.76583 (4)	0.05281 (16)	
Cl1	−0.25879 (14)	−0.40473 (13)	0.99518 (12)	0.0730 (3)	
Cl2	0.13155 (14)	0.78058 (13)	0.40236 (15)	0.0818 (4)	
O1	−0.2370 (3)	−0.1702 (3)	0.8662 (3)	0.0610 (7)	
O2	0.1088 (4)	0.4605 (3)	0.3572 (3)	0.0714 (8)	
C1	0.3337 (4)	0.0067 (4)	0.8013 (3)	0.0394 (8)	
C2	0.1712 (4)	0.0498 (4)	0.7722 (4)	0.0440 (8)	
H21	0.1548	0.1271	0.7328	0.053*	
C3	0.0353 (4)	−0.0224 (4)	0.8020 (3)	0.0423 (8)	
H31	−0.0729	0.0071	0.7825	0.051*	
C4	0.0557 (4)	−0.1385 (3)	0.8605 (3)	0.0362 (7)	
C5	0.2200 (4)	−0.1800 (4)	0.8892 (3)	0.0392 (8)	
H51	0.2365	−0.2574	0.9285	0.047*	
C6	0.3576 (4)	−0.1082 (4)	0.8603 (3)	0.0406 (8)	
H61	0.4661	−0.1368	0.8803	0.049*	
C7	0.4308 (4)	0.2302 (4)	0.6692 (4)	0.0415 (8)	
C8	0.3485 (5)	0.1721 (4)	0.5489 (4)	0.0488 (9)	
H81	0.3415	0.0695	0.5139	0.059*	
C9	0.2762 (5)	0.2656 (4)	0.4798 (4)	0.0470 (8)	
H91	0.2200	0.2256	0.3989	0.056*	
C10	0.2872 (4)	0.4188 (4)	0.5306 (3)	0.0402 (8)	
C11	0.3757 (5)	0.4760 (4)	0.6494 (4)	0.0490 (9)	
H111	0.3879	0.5787	0.6829	0.059*	
C12	0.4456 (5)	0.3831 (4)	0.7184 (4)	0.0500 (9)	
H121	0.5033	0.4232	0.7989	0.060*	
C13	−0.0979 (4)	−0.2116 (4)	0.8894 (3)	0.0425 (8)	
C14	−0.0706 (5)	−0.3401 (4)	0.9497 (4)	0.0514 (9)	
H141	0.0117	−0.3087	1.0244	0.062*	
H142	−0.0248	−0.4214	0.8898	0.062*	
C15	0.1994 (4)	0.5136 (4)	0.4539 (4)	0.0465 (9)	
C16	0.2244 (5)	0.6811 (4)	0.5070 (4)	0.0540 (10)	
H161	0.3444	0.7076	0.5243	0.065*	
H162	0.1758	0.7101	0.5872	0.065*	

### Atomic displacement parameters (Å^2^)

**Table:**

	*U*^11^	*U*^22^	*U*^33^	*U*^12^	*U*^13^	*U*^23^
Se1	0.03407 (19)	0.0680 (3)	0.0687 (3)	−0.00036 (16)	0.00438 (18)	0.0409 (2)
Cl1	0.0585 (6)	0.0786 (7)	0.0896 (9)	−0.0193 (5)	0.0163 (6)	0.0345 (6)
Cl2	0.0582 (6)	0.0802 (7)	0.1284 (12)	0.0049 (5)	0.0010 (7)	0.0700 (8)
O1	0.0318 (12)	0.0782 (19)	0.083 (2)	0.0059 (12)	0.0080 (13)	0.0385 (16)
O2	0.081 (2)	0.0686 (19)	0.060 (2)	0.0050 (16)	−0.0141 (17)	0.0170 (16)
C1	0.0312 (15)	0.0460 (18)	0.044 (2)	0.0008 (13)	0.0040 (15)	0.0170 (16)
C2	0.0387 (17)	0.0477 (19)	0.053 (2)	0.0035 (15)	0.0039 (16)	0.0268 (17)
C3	0.0326 (15)	0.0475 (19)	0.050 (2)	0.0065 (14)	0.0018 (15)	0.0183 (17)
C4	0.0303 (15)	0.0424 (17)	0.0371 (19)	0.0027 (13)	0.0037 (14)	0.0124 (15)
C5	0.0334 (15)	0.0426 (18)	0.048 (2)	0.0066 (13)	0.0045 (15)	0.0242 (16)
C6	0.0277 (14)	0.0510 (19)	0.048 (2)	0.0090 (14)	0.0006 (14)	0.0218 (16)
C7	0.0338 (16)	0.0500 (19)	0.046 (2)	−0.0004 (14)	0.0077 (16)	0.0214 (17)
C8	0.055 (2)	0.0373 (18)	0.054 (2)	−0.0036 (16)	0.0018 (19)	0.0130 (17)
C9	0.0465 (19)	0.050 (2)	0.043 (2)	−0.0039 (16)	−0.0016 (17)	0.0108 (17)
C10	0.0383 (17)	0.0430 (18)	0.044 (2)	−0.0007 (14)	0.0103 (16)	0.0178 (16)
C11	0.061 (2)	0.0396 (19)	0.045 (2)	−0.0020 (17)	0.0022 (19)	0.0107 (17)
C12	0.054 (2)	0.055 (2)	0.040 (2)	−0.0037 (18)	−0.0008 (18)	0.0127 (18)
C13	0.0337 (16)	0.053 (2)	0.042 (2)	−0.0001 (14)	0.0036 (15)	0.0146 (17)
C14	0.0443 (19)	0.057 (2)	0.059 (3)	−0.0034 (17)	0.0112 (19)	0.0253 (19)
C15	0.0394 (17)	0.054 (2)	0.053 (2)	0.0040 (16)	0.0098 (18)	0.0240 (19)
C16	0.047 (2)	0.049 (2)	0.077 (3)	0.0029 (16)	0.009 (2)	0.036 (2)

### Geometric parameters (Å, º)

**Table:**

Se1—C7	1.919 (3)	C7—C8	1.377 (5)
Se1—C1	1.920 (3)	C7—C12	1.383 (5)
Cl1—C14	1.766 (4)	C8—C9	1.383 (5)
Cl2—C16	1.757 (4)	C8—H81	0.9300
O1—C13	1.203 (4)	C9—C10	1.387 (5)
O2—C15	1.202 (5)	C9—H91	0.9300
C1—C6	1.384 (5)	C10—C11	1.382 (5)
C1—C2	1.392 (4)	C10—C15	1.495 (5)
C2—C3	1.371 (5)	C11—C12	1.372 (5)
C2—H21	0.9300	C11—H111	0.9300
C3—C4	1.388 (5)	C12—H121	0.9300
C3—H31	0.9300	C13—C14	1.510 (5)
C4—C5	1.398 (4)	C14—H141	0.9700
C4—C13	1.487 (4)	C14—H142	0.9700
C5—C6	1.376 (4)	C15—C16	1.517 (5)
C5—H51	0.9300	C16—H161	0.9700
C6—H61	0.9300	C16—H162	0.9700
			
C7—Se1—C1	100.05 (14)	C11—C10—C9	118.8 (3)
C6—C1—C2	120.0 (3)	C11—C10—C15	123.3 (3)
C6—C1—Se1	117.0 (2)	C9—C10—C15	117.9 (3)
C2—C1—Se1	123.0 (3)	C12—C11—C10	120.8 (3)
C3—C2—C1	119.7 (3)	C12—C11—H111	119.6
C3—C2—H21	120.2	C10—C11—H111	119.6
C1—C2—H21	120.2	C11—C12—C7	120.4 (4)
C2—C3—C4	121.5 (3)	C11—C12—H121	119.8
C2—C3—H31	119.2	C7—C12—H121	119.8
C4—C3—H31	119.2	O1—C13—C4	121.8 (3)
C3—C4—C5	118.0 (3)	O1—C13—C14	121.6 (3)
C3—C4—C13	118.3 (3)	C4—C13—C14	116.7 (3)
C5—C4—C13	123.7 (3)	C13—C14—Cl1	112.6 (3)
C6—C5—C4	121.1 (3)	C13—C14—H141	109.1
C6—C5—H51	119.4	Cl1—C14—H141	109.1
C4—C5—H51	119.4	C13—C14—H142	109.1
C5—C6—C1	119.7 (3)	Cl1—C14—H142	109.1
C5—C6—H61	120.1	H141—C14—H142	107.8
C1—C6—H61	120.1	O2—C15—C10	121.9 (3)
C8—C7—C12	119.4 (3)	O2—C15—C16	121.3 (4)
C8—C7—Se1	120.7 (3)	C10—C15—C16	116.7 (3)
C12—C7—Se1	119.9 (3)	C15—C16—Cl2	112.6 (3)
C7—C8—C9	120.2 (3)	C15—C16—H161	109.1
C7—C8—H81	119.9	Cl2—C16—H161	109.1
C9—C8—H81	119.9	C15—C16—H162	109.1
C8—C9—C10	120.4 (4)	Cl2—C16—H162	109.1
C8—C9—H91	119.8	H161—C16—H162	107.8
C10—C9—H91	119.8		
			
C6—C1—C2—C3	−0.2 (5)	C15—C10—C11—C12	176.3 (3)
Se1—C1—C2—C3	−179.2 (3)	C10—C11—C12—C7	1.0 (6)
C1—C2—C3—C4	−0.1 (6)	C8—C7—C12—C11	1.5 (5)
C2—C3—C4—C5	0.3 (5)	Se1—C7—C12—C11	179.0 (3)
C2—C3—C4—C13	−179.8 (3)	C3—C4—C13—O1	−1.9 (5)
C3—C4—C5—C6	0.0 (5)	C5—C4—C13—O1	178.0 (4)
C13—C4—C5—C6	−180.0 (3)	C3—C4—C13—C14	178.4 (3)
C4—C5—C6—C1	−0.3 (5)	C5—C4—C13—C14	−1.7 (5)
C2—C1—C6—C5	0.4 (5)	O1—C13—C14—Cl1	−6.1 (5)
Se1—C1—C6—C5	179.4 (3)	C4—C13—C14—Cl1	173.6 (3)
C12—C7—C8—C9	−2.2 (5)	C11—C10—C15—O2	−172.1 (4)
Se1—C7—C8—C9	−179.7 (3)	C9—C10—C15—O2	6.9 (5)
C7—C8—C9—C10	0.5 (5)	C11—C10—C15—C16	5.5 (5)
C8—C9—C10—C11	1.9 (5)	C9—C10—C15—C16	−175.5 (3)
C8—C9—C10—C15	−177.1 (3)	O2—C15—C16—Cl2	−7.9 (5)
C9—C10—C11—C12	−2.7 (5)	C10—C15—C16—Cl2	174.6 (2)

### Hydrogen-bond geometry (Å, º)

Cg1 and Cg2 are the centroids of benzene rings C1-C6 and C7-C12, respectively.

**Table:**

*D*—H···*A*	*D*—H	H···*A*	*D*···*A*	*D*—H···*A*
C6—H61···O1^i^	0.93	2.41	3.288 (4)	158
C16—H161···*Cg*2^ii^	0.97	2.82	3.611 (4)	140
C16—H162···*Cg*1^iii^	0.97	2.92	3.749 (4)	144

Symmetry codes: (i) *x*+1, *y*, *z*; (ii) −*x*+1, −*y*+1, −*z*+1; (iii) *x*, *y*+1, *z*.
