# Supplementary figures and images for: Crystal structure of 1,1′-[selanediyl­bis(4,1-phenyl­ene)]bis­(2-chloro­ethan-1-one)
Source: Acta Crystallogr E Crystallogr Commun. 2015 Nov 14;71(Pt 12):o935–6. doi: 10.1107/S2056989015019969 (PMC4719889; doi:10.1107/S2056989015019969)

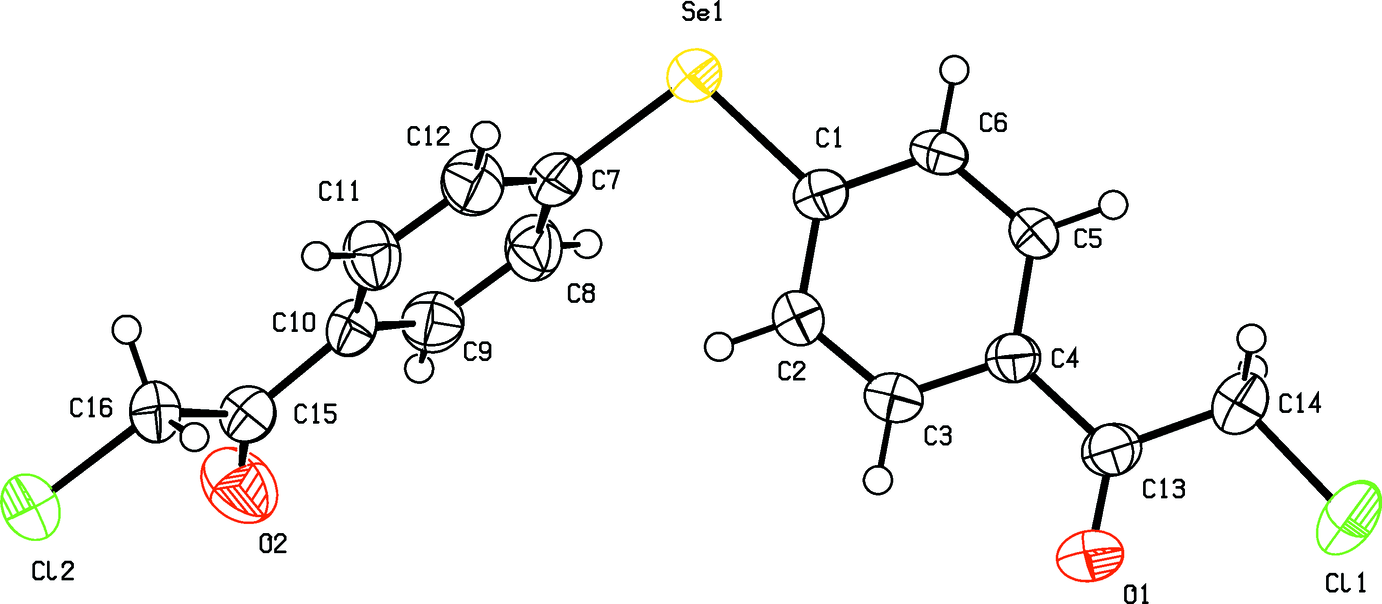

Supplement: Supplementary file 4 [file e-71-0o935-fig1.tif]

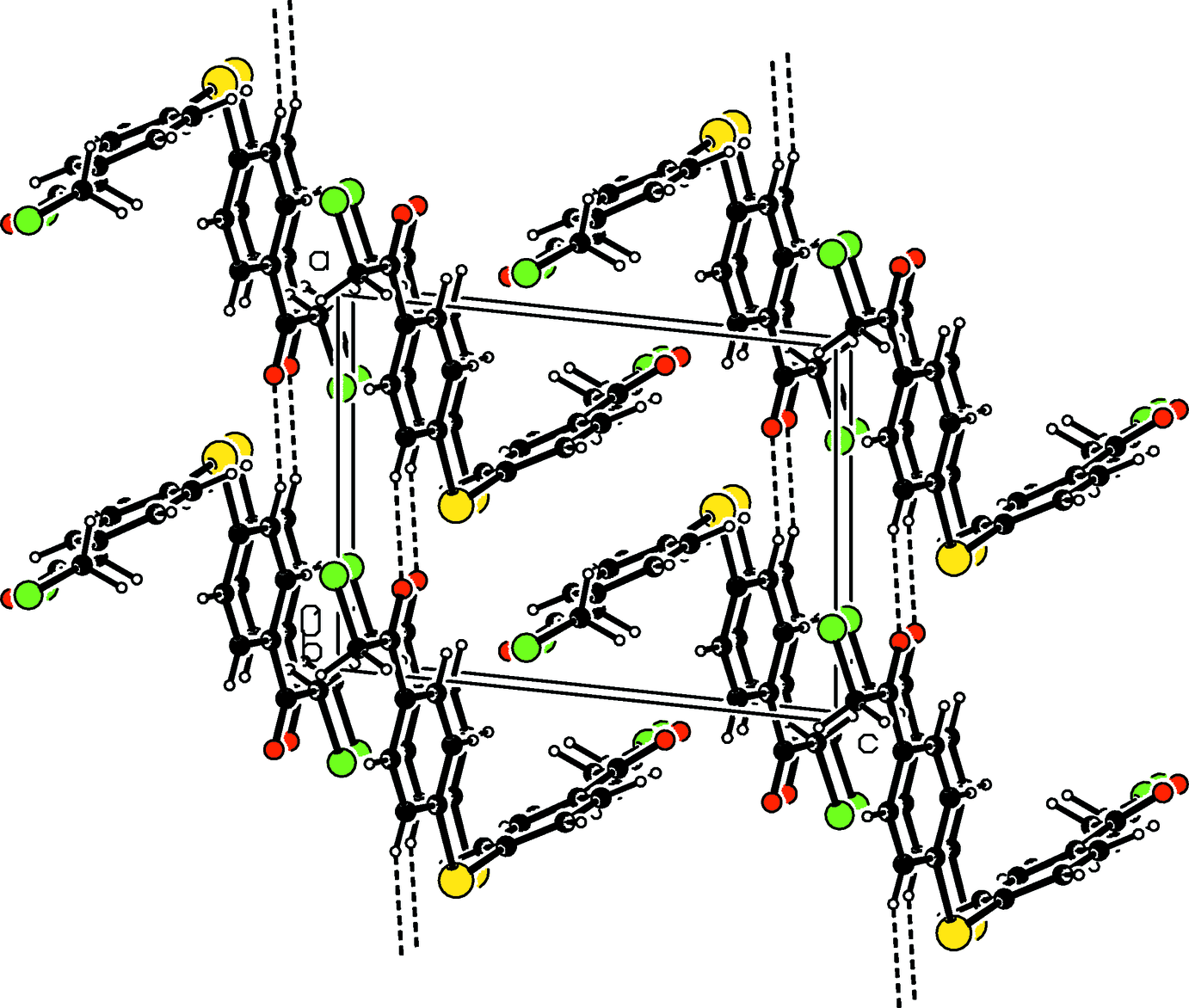

Supplement: Supplementary file 5 [file e-71-0o935-fig2.tif]
